# Morphology and Distribution of Fat Globules in Osteomyelitis on Magnetic Resonance Imaging

**DOI:** 10.2174/0115734056331041250116092101

**Published:** 2025-01-27

**Authors:** Li-Yuan Xie, Lei Cao, Wen-Juan Wu, Ji-Cun Liu, Na Zhao, Yong-Li Zheng, Xiao-Na Zhu, Bu-Lang Gao, Gui-Fen Han

**Affiliations:** 1 Department of Radiology, Hebei Provincial Luquan Prison Hospital, Shijiazhuang, China; 2 Department of Radiology, Hebei Medical University Third Hospital, Shijiazhuang, China; 3 Department of Radiology, The First Outpatient Department of Hebei Provincial Government Offices, Shijiazhuang China

**Keywords:** Osteomyelitis, Fat globules, Magnetic resonance imaging, Morphology, Distribution

## Abstract

**Introduction:**

The purpose of this study was to investigate the morphology and distribution characteristics of fat globules in osteomyelitis on magnetic resonance imaging (MRI).

**Materials and Methods:**

Patients with pathologically-confirmed osteomyelitis and MRI scans were retrospectively enrolled, and fat globules on the MRI images were analyzed.

**Results:**

Among 103 patients with non-traumatic osteomyelitis, 75 were fat globule negative and 28 were positive. There was no statistically significant difference in age and gender between patients with and without fat globules (*p*>0.05). The inflammatory indicators (CRP, ESR, WBC, and NEUT) in the fat globule positive group were significantly higher (*p*<0.05) than those in the negative group. The lesions were mainly located in the long bones of the limbs in patients with positive fat globules. Twenty-eight patients (27.2% or 28/103) were detected to have fat globules on MRI images, including 20 males (71%) and 8 females (29%) aged 5-64 years (mean 16 years). The time from onset to MRI examination was 8 days to 4 months. The location of fat globules was in the tibia in 10 patients (35.7%), femur in 8 (28.6%), humerus in 4 (14.3%), radius in 2 (7.1%), ulna in 1 (3.6%), calcaneus in 1 (3.6%), sacrum in 1 (3.6%), and fibula in 1 patient (3.6%). On MRI imaging, 28 cases (100%) showed widely distributed patches or tortuous and sinuous abnormal signals in the bone marrow. In 25 cases (89.2%), a grid-like abnormal signal was found in the subcutaneous soft tissue. In 21 patients (75%), pus was found in the adjacent extraosseous soft tissues. Among 28 patients with fat globules, 17 patients (60.7%) had fat globules only in the adjacent extraosseous soft tissue, 6 patients (21.4%) had only intraosseous fat globules (including 5 cases with halo signs around the fat globules and 1 case (3.6%) with fat globules located at the edge of the pus cavity inside the bone without a halo sign), and 5 patients (17.8%) had both intraosseous and extraosseous fat globules. Of 6 patients (21.4% or 6/28) with liquid levels, the liquid level appeared outside the bone.

**Conclusion:**

The appearance of fat globules on MRI in patients with osteomyelitis indicates severe infection. Fat globules of osteomyelitis may present with diverse shapes inside and outside the bone marrow as one of the MRI signs of osteomyelitis, with a probability of approximately 27.2%. They have high specificity in diagnosing osteomyelitis and can be used for diagnosis and differential diagnosis.

## 1 INTRODUCTION

Fat globules are thought to be of high signals on magnetic resonance imaging (MRI) that are close to or higher than the signal of subcutaneous fat when a patient develops osteomyelitis, and the high signals can be completely suppressed in the MRI fat suppression sequence [1-4]. The morphology of fat globules is at least 2 millimeters in diameter in a singular occurrence or multiple occurrences at least on two MRI imaging planes [3, 4]. Fat globules are a more specific sign of osteomyelitis [5-9]. At present, reports on MRI signs of osteomyelitis mainly focus on describing changes in bone and soft tissue signals, bone destruction, and abscesses [1, 10]. It is well known that some bone tumors, such as Ewing's sarcoma, may be similar to osteomyelitis in both clinical (both may present with fever, increased serum inflammatory markers, and pain) and imaging manifestations (invasive periosteal reactions, cortical destruction, and joint fatigue) [8, 11], making it crucial to distinguish osteomyelitis from malignant tumors in a timely and accurate manner because the treatment approaches and outcomes are completely different [8, 12]. In previous studies, intramedullary or extramedullary fat globules have also been identified as specific signs of osteomyelitis, and these signs are also thought to potentially help distinguish osteomyelitis from malignancy [7]. However, there are no relevant articles that provide a detailed summary of the morphology and distribution characteristics of fat globules on MRI images of osteomyelitis, and many radiologists cannot accurately identify fat globules. We retrospectively collected a dataset of patients with osteomyelitis confirmed by surgery and pathology, and analyzed the MRI morphology and distribution characteristics of fat globules associated with osteomyelitis. The purpose of this study was to further improve radiologists' understanding of fat globules in osteomyelitis and provide more imaging bases for clinicians to accurately diagnose osteomyelitis.

## 2 MATERIALS AND METHODS

This retrospective one-center study was performed on humans according to the Helsinki Declaration of 1975, as revised in 2013 (http://ethics.iit.edu/ecodes/node/3931), after approval by the ethics committee of the Third Hospital of Hebei Medical University, China (W2020-059-1). Informed consent was waived by the same ethics committee of the Third Hospital of Hebei Medical University because of the retrospective study design. All methods were conducted in accordance with relevant guidelines and regulations. Patients with non-traumatic osteomyelitis confirmed by surgery and pathology who had undergone MRI were retrospectively enrolled. The inclusion criteria were patients with non-traumatic osteomyelitis and fat globules confirmed by surgery and pathology who had undergone an MRI scan. Postoperative pathological examination was performed on all diseased bone tissues, and bacterial culture and drug sensitivity tests were performed on the concentrated solution.

MRI examination was conducted using the German Siemens 1.5T Avanto MR scanner in the cross-sectional, coronal, and sagittal scans. The scanning layer thickness was 3.0 mm, and the interlayer spacing was 4.0 mm. The field of view (FOV) varied depending on the extent of the lesion. The scanning sequence included the following: (1) cross-sectional spin echo sequence (SE) T1WI, TR 512.0-550.0 ms, and TE 8.4-11.0 ms; (2) short time reversal recovery sequence (STIR) T2WI, TR 3 700.0 ms, TE 38.0 ms, and TI 160.0 ms; (3) sagittal plane spin echo sequence (SE) T1WI, TR 512.0-550.0 ms, and TE8.4-11.0 ms; (4) sagittal fat suppression fast spin echo sequence (FS-TSE) T2WI, TR 3000.0-3500.0 ms, and TE 40.0-64.0 ms; (5) coronal fat suppression fast spin echo sequence (TSE) T2WI, TR 3800.0-4300.0 ms, and TE 106.0-108.0 ms.

MRI image analysis was conducted by one chief physician (with 25 years of experience) and one attending physician independently (with 12 years of experience). If the results were inconsistent, a consensus diagnosis was reached through consultation or with the involvement of a third senior physician. Comparative analysis was conducted of age, sex, C-reactive protein (CRP), erythrocyte sedimentation rate (ESR), white blood cell count (WBC), and neutrophils (NEUT) between two groups of osteomyelitis patients (fat globule positive and fat globule negative groups). In analyzing the MRI imaging manifestations of osteomyelitis, the focus was on observing the distribution, number, size, and morphology of fat globules. In measuring fat globules, the size of a single fat globule was measured by its length and diameter, whereas the size of multiple fat globules was measured by the meridian length of the smallest and largest fat globules, respectively.

### 2.1 Statistical Analysis

The statistical analysis was performed using the SPSS 20 software (IBM, Chicago, IL, USA). Continuous measurement data have been expressed as mean and standard deviation or median and interquartile range, with the inter-group comparison performed using the Wilcoxon rank sum test. Categorical data have been presented as frequency and percentage, and comparison analysis between groups was conducted using the Chi-square test. *p*<0.05 was considered statistically significant for the difference.

## 3 RESULTS

One hundred and three patients with non-traumatic osteomyelitis were retrospectively enrolled, with 75 patients having negative fat globules and 28 patients (27.2% or 28/103) having fat globules on MRI images (Tables **1** and **2** and Figs. (**1**-**3**)). Comparative analysis was performed of age, CRP, ESR, WBC, and NEUT between patients with positive and negative fat globules (Table **3**). The levels of CRP, ESR, WBC, and NEUT were significantly higher (*p*<0.05) in the fat globule positive group than in the fat globule negative group, whereas no significant (*p*>0.05) difference was found in age and sex between the two groups (*p* >0.05).

Among the 28 patients with positive fat globules, there were 20 males (71%) and 8 females (29%) with an age range of 5-64 years (mean 16 years). The time from onset to MRI examination was 8 days to 4 months (mean 21 days). The location of the fat globules was in the tibia in 10 patients (35.7%), femur in 8 (28.6%), humerus in 4 (14.3%), radius in (7.1%), ulna in 1 (3.6%), calcaneus in 1 (3.6%), sacrum in 1 (3.6%), and fibula in 1 patient (3.6%) (Fig. **4**). Twenty-six cases (92.8%) underwent osteomyelitis lesion clearance and vacuum sealing drainage (VSD) negative pressure suction surgery, 1 case (3.6%) underwent abscess puncture and fluid extraction under ultrasound guidance, and 1 case (3.6%) underwent osteomyelitis lesion clearance and bone cement implantation surgery.

In pus culture and antimicrobial susceptibility test, 4 patients (14.3% or 4/28) had no bacterial growth, 22 cases (78.6% or 22/28) were infected with *Staphylococcus aureus*, 1 case (3.6% or 1/28) was detected to have infection of methicillin-resistant *Staphylococcus aureus* (MRSA), and 1 case (3.6% or 1/28) was infected with *Enterococcus faecalis* (blood culture was positive with *Staphylococcus aureus*).

**Fig. (1):**
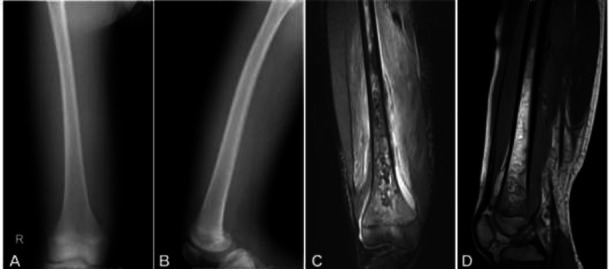
X-ray angiograms and magnetic resonance imaging (MRI) of the right femur. (**A** & **B**). Anteroposterior and lateral X-ray angiograms showed intact bone structure of the femur, without abnormal bone density, but with thickening of soft tissue and increased density of subcutaneous fat layer, which had a blurred boundary with muscles. (**C** & **D**). Coronal fat suppression (FS) MRI T2WI (**C**) and sagittal T1WI (**D**) of the right femur demonstrated patchy and tortuous mixed abnormal signals within the femoral bone marrow cavity and uneven and thinning cortical destruction in the lower femur. Diffuse high uneven signal shadows were shown within and between the muscles and in the subcutaneous fat layer in the right thigh. Abnormal grid-like signals were observed in the subcutaneous fat layer, and pus was revealed to surround the femur as high fluid signals.

In the analysis of X-ray plain film images, 18 cases (64.3% or 18/28) showed irregular destruction and thinning of the bone cortex, 1 patient (3.6% or 1/28) had severe destruction of the bone cortex and disappearance of the normal bone cortex contour, and 9 patients (32.1% or 9/28) had normal bone morphology (Fig. **1**).

On MRI imaging, 28 cases (100%) showed widely distributed patches or tortuous and sinuous abnormal signals in the bone marrow. Compared to adjacent normal bone marrow signals, the T1WI signal was reduced, and the lesions on the fat inhibition T2WI or STIR sequences were more clearly displayed (Fig. **1**). In all 28 cases (100%), T1WI hypointensity and T2WI hyperintensity signals were found in the periosseous soft tissues, and these abnormalities were distributed in and between muscles, with blurred boundaries. In 25 cases (89% or 25/28), a grid-like abnormal signal was found in the subcutaneous soft tissue, indicating soft tissue swelling. In 21 patients (75% or 21/28), pus was found in the adjacent extraosseous soft tissues, and the pus signal was like a liquid with T1WI low intensity and T2WI high-intensity signals.

**Fig. (2):**
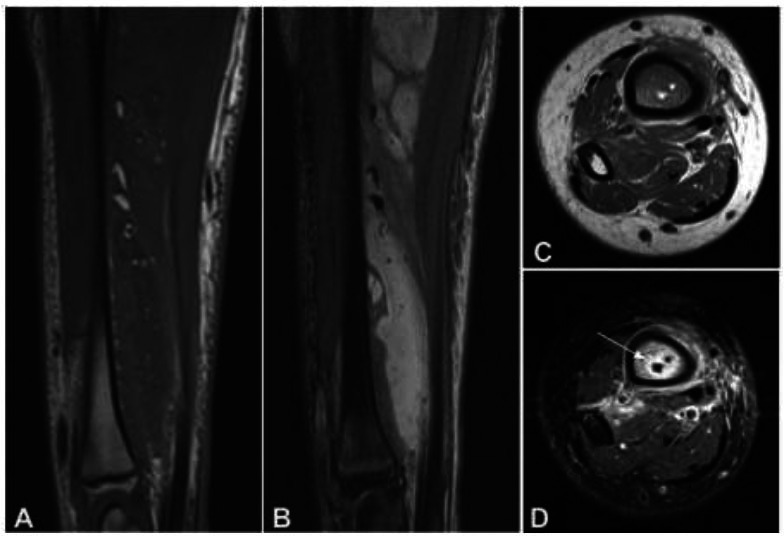
Magnetic resonance imaging (MRI). (**A** & **B**). Ulna MRI imaging was shown. (**A**). MRI sagittal T1WI showed diffuse low signal shadows in the forearm soft tissue, with scattered punctate (some lesions less than 2mm) and small circular high signal shadows inside. The signal was higher than that of normal yellow bone marrow, similar to the subcutaneous fat signal. (**B**). MRI sagittal fat suppression (FS) T2WI demonstrated diffuse high signal intensity in the forearm soft tissue, with punctate and small circular high-intensity signals on T1WI and low-intensity signals on fat suppression T2WI (similar to fat signal intensity). These abnormally distributed fat signals were scattered in a large amount of high-signal pus around the ulna. C & D. MRI plain cross-section T1WI (**C**) and cross-sectional short time reversal recovery sequence (STIR) T2WI (**D**) revealed small circular and irregular patchy high signal intensities on T1WI in the medulla (**C**). The signal on T2WI (STIR) (**D**) was low, surrounded by a high signal halo sign (arrow).

**Fig. (3):**
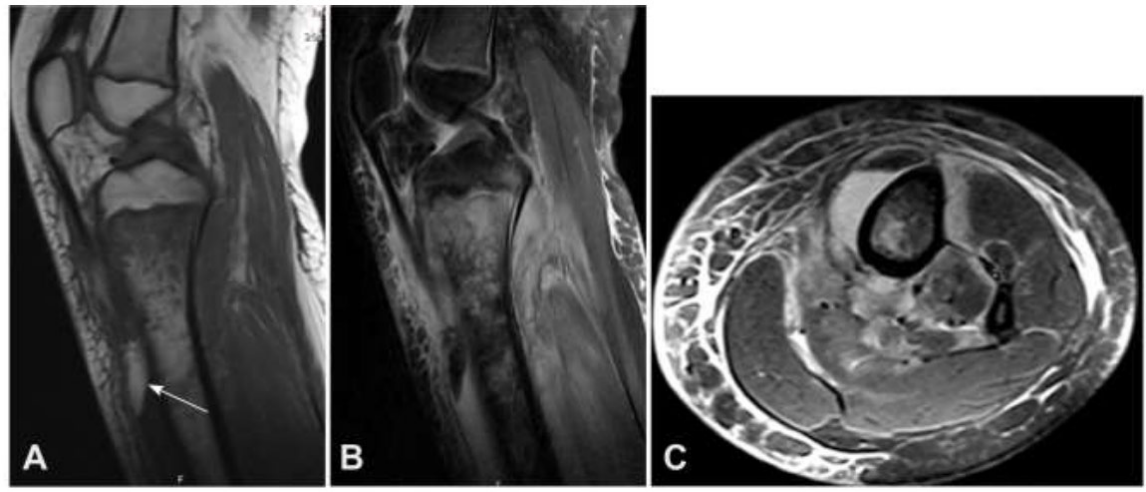
Magnetic resonance imaging (MRI) of the fluid level. (**A** & **B**). MRI sagittal T1WI (**A**) and fat suppression (FS) T2WI (**B**) demonstrated the lipid level (arrow) in the front of the upper segment of the tibia, with T1WI showing an equal signal between the lower part of the fluid level and the muscle, and a high signal similar to fat in the upper part. C. MRI plain scan cross-sectional short-term reversal recovery sequence (STIR) T2WI showed a high signal similar to liquid in the lower part and a low signal in the upper part.

**Fig. (4):**
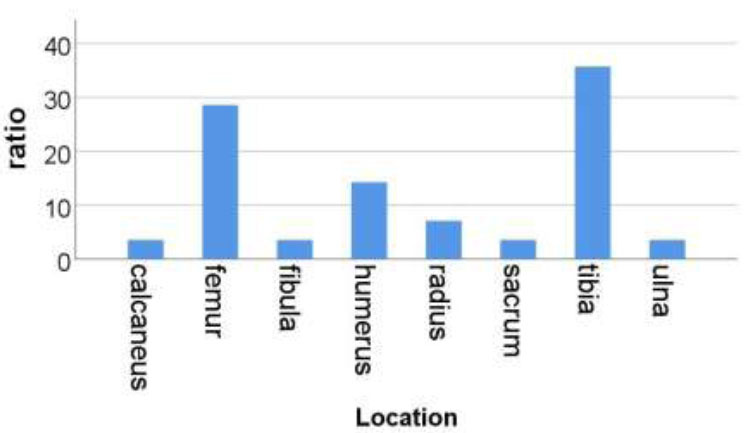
Location of lesion.

In the analysis of the appearance of fat globules, multiple fat globules were present in 16 patients (57.1%), and a single fat globule in 12 (42.9%). Seventeen patients (60.7% or 17/28, cases 6-22; Table **1**) (3 with lipid-liquid levels) only had fat globules in the adjacent extraosseous soft tissue, without fat globules inside the bone (Fig. **2**). Six patients (21.4% or 8/28, cases 23-28; Table **1**) had fat globules only inside the bone, including 5 cases with halo signs around the fat globules (Fig. **2**) and 1 case (3.6% or 1/28) with fat globules located at the edge of the pus cavity inside the bone without a halo sign. Five patients (17.9% or 9/28, cases 1-5; Table **1**) had both intraosseous and extraosseous fat globules, and two patients had lipid-liquid levels. The size of the fat globules ranged from 0.83-18.7 mm (mean 7.5 mm). Of the 5 patients (17.9% or 5/28) with liquid levels, the liquid level appeared outside the bone (Fig. **3** and Table **1**), with the maximal diameter of the liquid level being 6.3-29.8 mm (mean 14.5 mm).

## 4 DISCUSSION

In this study investigating the morphology and distribution characteristics of fat globules in osteomyelitis on MRI images, it was found that fat globules of osteomyelitis may present with diverse shapes inside and outside the bone marrow as one of the MRI signs of osteomyelitis, with a probability of approximately 27.2%. The fat globules were mainly located in the long bones of the limbs, especially in the femur and tibia. The inflammatory indicators (CRP, ESR, WBC, and NEUT) in the fat globule positive group were significantly higher (*p*<0.05) than in the fat globule negative group, indicating that patients with fat globules may have more severe conditions than those without fat globules. Based on this finding, treatment should be more proactive. Therefore, when osteomyelitis occurs in the long bones of limbs, radiologists should carefully observe whether there are fat globules inside or outside the bone marrow on MRI examination.

MRI has a high tissue resolution in multiple directions and high sensitivity (82%-100%) and specificity (38%-100%) in diagnosing osteomyelitis [13, 14]. Therefore, it is the best imaging examination method for early osteomyelitis. The main MRI signs of osteomyelitis are patchy abnormal signals in the bone marrow. Compared to adjacent normal bone marrow signals, the T1WI signal is reduced, the T2WI signal is increased, and the lesions are more clearly displayed on sequences, such as fat suppression T2WI or STIR. The soft tissues around the bone cortex show diffused distribution of T1WI low signal and T2WI high signal shadows, with blurred boundaries. As the disease progresses, periosteal reactions, irregular destruction of the bone cortex, and abscess formation may appear on MRI [2, 13-17]. All patients with osteomyelitis in this study had patchy abnormal signals on T1WI, fat-suppressed T2WI, or STIR in the soft tissues inside and outside the bone. Nineteen patients had bone destruction, and 16 patients had abscesses in the soft tissues around the bone. These abnormal MRI signs are common non-specific signs of osteomyelitis.

On MRI, fat globules are presented as a high signal lesion close to or higher than the signal of subcutaneous fat, and the signal on the fat suppression sequence can be completely suppressed. Fat globules can be detected on at least two imaging planes. In liquid-sensitive sequences (such as fat suppression T2WI and fat suppression proton density imaging), there may be a high signal halo around some fat globules, known as the halo sign [3]. The fat globules of 28 patients in this group were consistent with the MRI findings of fat globules on T1WI and fat suppression T2WI. Among them, the fat globules of 5 patients with lesions limited to non-purulent intramedullary cavities were all haloed on fat suppression T2WI or STIR. It is suggested that the halo sign may be related to the inflammatory response of necrotic fat lesions, similar to those seen in necrotic subcutaneous tissues of limbs and trunk, as well as fat necrosis in the breast [3, 18-20].

In morphology, fat globules mainly manifest as round, patchy, and lipid-liquid levels, formed by the coexistence of fat globules and pus in osteomyelitis [2, 5, 6, 9]. The diameter of fat globules was believed to be at least 2 millimeters in single or multiple occurrences [3]. In this group of cases, the smallest measurable fat globule diameter on MRI images of patients with osteomyelitis of the radius and ulna was about 0.8 millimeters. In addition, in 7 patients, fat globules with a diameter less than 2.0 millimeters were also measurable on the images. Among them, one patient had multiple punctate fat globules in the soft tissue that could not be measured due to the small size of the lesion (Fig. **2**). Among 28 patients, 12 had single fat globules, whereas the other 16 had multiple ones. We believe that as long as the lesion is determined to be entirely composed of adipose tissue rather than yellow bone marrow, it can be clearly diagnosed as a fat globule, without the need to define its diameter as at least 2 millimeters large. In addition, fat globules can occur inside and outside the medulla [2, 7]. In this group, the fat globules were limited to the medulla in 6 cases, outside the medulla in 17 cases, and both inside and outside the medulla in 5 cases.

For the mechanism, fat globules are believed to be an inflammatory response of osteomyelitis that causes congestion, exudation, and edema in the bone marrow cavity, ultimately leading to increased intramedullary pressure. A large number of fat cells (*i.e*., adipocytes) in the marrow cavity rapidly necrotize, release free lipids, and aggregate to form fat globules [2, 7]. The formation of fat globule sign is the necrosis of a large number of myeloid fat cells in a short time, rather than the necrosis of a single lipoid marrow cell. However, in some aspects, this phenomenon is different from that of traditional fat necrosis, because there are no foam macrophages associated with the assumed fat globule in the bone tissue of the lesion area [7]. The histological changes in bone marrow cavities in patients with osteomyelitis confirm the theory that damaged bone tissue releases fat globules [7]. In this group of cases, there were a total of 27 cases where fat globules were located inside the medulla and around the bone. The location of the lesions in these 27 cases indirectly proves that fat globules are closely related to bone tissue, and are far away from subcutaneous tissue rich in fat tissue. When the bone cortex is damaged, the already free lipids can enter the soft tissue outside the bone through the damaged bone cortex area, so the appearance of fat globules or lipid levels outside the bone indicates that the bone cortex has been damaged [5, 21]. In 7 patients whose bone cortex was not damaged on X-ray plain films in our study, fat globules appeared around the bones on MRI images. Therefore, it can be inferred that the bone cortex of these patients has actually been damaged, but these bone cortex damages cannot be shown on X-ray plain films yet.

The fat globule sign has important clinical significance. Firstly, fat globules help in diagnosis and differential diagnosis. Some tumors, such as Ewing's sarcoma, have very similar clinical manifestations, blood routine examination, and X-ray plain film or CT examination manifestations to those of osteomyelitis, and sometimes it is difficult to distinguish between the two. The appearance of fat globules suggests that the lesion is osteomyelitis rather than Ewing's sarcoma. Davies *et al*. reported that 12 out of 17 cases of osteomyelitis showed fat globular signs, suggesting that the presence of fat globular signs inside and outside the bone marrow on MRI is a specific sign of osteomyelitis, which may differentiate tumors from osteomyelitis [7]. However, some researchers believe that the fat globule sign is not a specific sign of osteomyelitis and needs to be distinguished from trauma or soft tissue masses containing lipid components, such as liposarcoma and dermoid cysts [21, 22]. However, based on medical history, clinical manifestations, laboratory tests, and other imaging signs, it is easy to distinguish osteomyelitis from these lesions. Moreover, fat globules indicate that the bone cortex has been damaged. When the fat globule sign appears in extramedullary soft tissue, it indicates that osteomyelitis has caused damage to the bone cortex [5], and fat globules may indirectly suggest bone destruction that cannot be detected by X-ray and CT examinations. In addition, fat globules are markers of increased intramedullary pressure and rapid necrosis of a large number of myeloid adipocytes. The results of this study showed that the inflammatory indicators (CRP, ESR, WBC, NEUT) in the fat globule positive group were significantly higher (*p*<0.05) than those in the fat globule negative group, indicating that the condition of fat globule positive patients was more severe. Therefore, doctors should be more proactive in their treatment. When osteomyelitis occurs in the long bones of limbs, fat globules should be carefully checked inside and outside the bone marrow during the patients' MRI examination.

Some limitations existed in this study, including the retrospective and one-center study design, a small cohort of sample, enrollment of Chinese patients, no control, and no randomization, which may all affect the generalization of the outcomes. Future prospective, randomized, controlled studies need to be performed with the involvement of multiple medical centers and races or ethnicities for better outcomes.

## CONCLUSION

To sum up, fat globules of osteomyelitis may present with diverse shapes inside and outside the bone marrow as one of the MRI signs of osteomyelitis, with a probability of approximately 27.2%. They have high specificity in diagnosing osteomyelitis and can be used for diagnosis and differential diagnosis.

## Figures and Tables

**Table 1:** Information related to patients with fat globules.

No./sex/age	Location	Multiple or Single	Morphology	Size (mm)	Halo Sign	Maximal Diameter of Liquid Level (mm)
1/F/12	Tibia (intra- and extraosseous)	Multiple	Round, strip, and liquid level	3.7-18.7	No	29.8
2/M/14	Femur (intra- and extraosseous)	Multiple	Strip and liquid level	5.9-7.9	No	6.3
3/F/10	Femur (intra- and extraosseous)	Multiple	Small round pieces	3.6-4.2	No	——
4/M/13	Humerus (intra- and extraosseous)	Multiple	Round and strips	1.7-16.8	Yes	——
5/M/9	Humerus (intra- and extraosseous)	Multiple	Round and strips	2.0-5.9	Yes	——
6/F/12	Calcaneum (extraosseous)	Single	Liquid level	3.0-8.9	No	7.4
7/M/14	Ulna (extraosseous)	Multiple	Small round pieces	1.6-8.9	No	——
8/F/13	Femur (extraosseous)	Multiple	Strips	1.7-12.0	No	——
9/F/8	Tibia (extraosseous)	Single	Liquid level	2.2-9.1	No	13.9
10/M/9	Tibia (extraosseous)	Multiple	Small round pieces	2.0-7.6	No	——
11F/12	Femur (extraosseous)	Single	Strips	16.7	No	——
12/M/14	Humerus (extraosseous)	Multiple	Round and strips	3.0-8.9	No	——
13/M/8	Femur (extraosseous)	Single	Strips	2.9	No	——
14/M/12	Tibia (extraosseous)	Single	Strips	7.8	No	——
15/M/12	Humerus (extraosseous)	Multiple	Round and strips	2.2-9.1	No	——
16/M/10	Tibia (extraosseous)	Single	Liquid level	7.87	No	15.9
17/M/11	Humerus (extraosseous)	Single	Strips	4.9	No	——
18/M/11	Radius (extraosseous)	Single	Round	5.2	No	——
19/M/12	Radius (extraosseous)	Multiple	Round	3.1-10.2	No	——
20/M/8	Femur (extraosseous)	Single	Strips	3.8	No	——
21/F/9	Femur (extraosseous)	Multiple	Round and strips	1.4-14.5	No	——
22/F/15	Fibula (extraosseous)	Multiple	Round and strips	1.9-7.3	No	——
23/M/5	Femur (intraosseous)	Single	Round	2.7	Yes	——
24/M/13	Tibia (intraosseous)	Multiple	Round and strips	0.8-8.3	Yes	——
25/M/63	Tibia (intraosseous)	Multiple	Small round pieces	2.8-7.5	Yes	——
26//M/49	Femur (intraosseous)	Multiple	Strips	1.7-8.1	Yes	——
27/M/64	Sacrum (intraosseous	Single	Round	3.2	Yes	——
28/M/13	Tibia (intraosseous pus cavity)	Single	Round	3.1	No	——

**Table 2:** Data of patients.

Variables		Data
Demography	No. of patients	28
	M/F	20/8
	Age (y, mean)	5-64 (16)
	Duration from onset to MRI scan (d, mean)	8-120 (21)
Fat globules location	Tibia	10 (35.7%)
	Femur	8 (28.6%)
	Humerus	4 (14.3%)
	Ulna	1 (3.6%)
	Calcaneus	1 (3.6%)
	Sacrum	1 (3.6%)
	Fibula	1 (3.6%)
Antimicrobial test	*Staphylococcus aureus*	22 (78.6%)
	Methicillin-resistant *Staphylococcus aureus*	1 (3.6%)
	*Enterococcus faecalis*	1 (3.6%)
	No bacteria	4 (14.3%)
Blood culture	*Staphylococcus aureus*	1 (3.6%)
plain X-ray imaging	Normal	9 (32.1%)
MRI imaging	Irregular bone destruction	18 (64.3%)
	Severe bone destruction	1 (3.6%)
	Abnormal signals in bone marrow	28 (100%)
Periosteous soft tissue	T1WI hypointensity signal	28 (100%)
	T2WI hyperintensity signal	28 (100%)
Fat globules	Extraosseous	17 (60.7%)
	Intraosseous	6 (21.4%)
	Both intra- and extraosseous	5 (17.9%)
	Size (mm)	0.83-18.7 (7.5)
	Single	12 (42.9%)
	Multiple	16 (57.1%)
Halo sign	-	6 (21.4%)
Liquid level	No. of patients	5 (17.9%)
	Maximal size (mm)	6.3-29.8 (14.5)

**Table 3:** Comparison between patients with positive and negative fat globules.

-	Positive	Negative	Z/X2	*p*-value
age	4-64 (mean13.14)	2-71 (mean19.92)	-1.245	0.213
CRP	39.04 (63.88)	13.07 (43.34)	-2.683	0.007
ESR	57.50 (48.00)	24.00 (42.00)	-3.955	0.000
WBC	11.61 (9.31)	6.88 (3.79)	-3.714	0.000
NEUT	72.45 (17.45)	59.20 (20.10)	-3.113	0.002
Gender	Male (n=20) Female (n=8)	Male (n=48) Female (n=27)	0.502	0.479

## Data Availability

All data generated or analyzed during this study are included in this published article.

## LIST OF ABBREVIATIONS

MRI: Magnetic Resonance Imaging
CRP: C-reactive protein
NEUT: Neutrophils
WBC: White Blood Cell Count
ESR: Erythrocyte Sedimentation Rate

## STANDARDS OF REPORTING

STROBE guidelines were followed.

## References

[1] Aydingoz U. (2023). Imaging osteomyelitis: An update.. Röfo Fortschr. Geb. Röntgenstr. Neuen Bildgeb. Verfahr..

[2] Godoy I.R.B., Neto L.P., Rodrigues T.C., Skaf A. (2018). Intra and extramedullary fat globules as an MRI marker for osteomyelitis.. Radiol. Case Rep..

[3] Robinson P., Farrant J.M., Bourke G., Merchant W., McKie S., Horgan K.J. (2008). Ultrasound and MRI findings in appendicular and truncal fat necrosis.. Skeletal Radiol..

[4] Wong A., Grando H., Fliszar E., Pathria M., Chang E.Y., Resnick D. (2014). Intramedullary fat globules related to bone trauma: A new MR imaging finding.. Skeletal Radiol..

[5] Hui C.L., Naidoo P. (2003). Extramedullary fat fluid level on MRI as a specific sign for osteomyelitis.. Australas. Radiol..

[6] Kumar J., Bandhu S., Kumar A., Alam S. (2007). Extra-osseous fat fluid level: A specific sign for osteomyelitis.. Skeletal Radiol..

[7] Davies A.M., Hughes D.E., Grimer R.J. (2005). Intramedullary and extramedullary fat globules on magnetic resonance imaging as a diagnostic sign for osteomyelitis.. Eur. Radiol..

[8] Kasalak Ö., Overbosch J., Adams H.J.A., Dammann A., Dierckx R.A.J.O., Jutte P.C., Kwee T.C. (2019). Diagnostic value of MRI signs in differentiating Ewing sarcoma from osteomyelitis.. Acta Radiol..

[9] Mattis T.A., Borders H.L., Ellinger D.M., Junewick J.J. (2011). Relationship between the clinical characteristics of osteomyelitis and the finding of extraosseous fat on MRI in pediatric patients.. Pediatr. Radiol..

[10] Crim J., Salmon S., Waranch C., Elfrink J., Layfield E., Stensby J.D. (2022). Update on MRI findings of osteomyelitis of long bones in the adult population.. Skeletal Radiol..

[11] Shimose S., Sugita T., Kubo T., Matsuo T., Nobuto H., Ochi M. (2008). Differential diagnosis between osteomyelitis and bone tumors.. Acta Radiol..

[12] Gaspar N., Hawkins D.S., Dirksen U., Lewis I.J., Ferrari S., Le Deley M.C., Kovar H., Grimer R., Whelan J., Claude L., Delattre O., Paulussen M., Picci P., Sundby Hall K., van den Berg H., Ladenstein R., Michon J., Hjorth L., Judson I., Luksch R., Bernstein M.L., Marec-Bérard P., Brennan B., Craft A.W., Womer R.B., Juergens H., Oberlin O. (2015). Ewing sarcoma: Current management and future approaches through collaboration.. J. Clin. Oncol..

[13] Song K.M., Sloboda J.F. (2001). Acute hematogenous osteomyelitis in children.. J. Am. Acad. Orthop. Surg..

[14] Woods C.R., Bradley J.S., Chatterjee A., Copley L.A., Robinson J., Kronman M.P., Arrieta A., Fowler S.L., Harrison C., Carrillo-Marquez M.A., Arnold S.R., Eppes S.C., Stadler L.P., Allen C.H., Mazur L.J., Creech C.B., Shah S.S., Zaoutis T., Feldman D.S., Lavergne V. (2021). Clinical practice guideline by the pediatric infectious diseases society and the infectious diseases society of america: 2021 guideline on diagnosis and management of acute hematogenous osteomyelitis in pediatrics.. J. Pediatric Infect. Dis. Soc..

[15] Weaver J.S., Omar I.M., Mar W.A., Klauser A.S., Winegar B.A., Mlady G.W., McCurdy W.E., Taljanovic M.S. (2022). Magnetic resonance imaging of musculoskeletal infections.. Pol. J. Radiol..

[16] Bury D.C., Rogers T.S., Dickman M.M. (2021). Osteomyelitis: Diagnosis and treatment.. Am. Fam. Physician.

[17] Pugmire B.S., Shailam R., Gee M.S. (2014). Role of MRI in the diagnosis and treatment of osteomyelitis in pediatric patients.. World J. Radiol..

[18] Walsh M., Jacobson J.A., Kim S.M., Lucas D.R., Morag Y., Fessell D.P. (2008). Sonography of fat necrosis involving the extremity and torso with magnetic resonance imaging and histologic correlation.. J. Ultrasound Med..

[19] Daly C.P., Jaeger B., Sill D.S. (2008). Variable appearances of fat necrosis on breast MRI.. AJR Am. J. Roentgenol..

[20] Chan P.Y.L., Wong T., Chau C.M., Fung W.Y., Lai K.B., Chan R.L.S., Wong W.C.W., Yung W.T., Ma J.K.F. (2023). Fat necrosis in the breast: A multimodality imaging review of its natural course with different aetiologies.. Clin. Radiol..

[21] Swain F.R., Strongwater A., Milman E. (2011). Diagnosis and triage of a patient with an extra-osseous fat fluid level.. Emerg. Radiol..

[22] Davis D.L., Vachhani P. (2015). Traumatic extra-capsular and intra-capsular floating fat: Fat-fluid levels of the knee revisited.. J. Clin. Imaging Sci..

